# Utility of the Phoenix-8 and PELOD-2 scales in pediatric patients with sepsis and acute lymphoblastic leukemia admitted to a pediatric intensive care unit at a quaternary-level hospital in Bogotá, Colombia, 2022–2024

**DOI:** 10.3389/fped.2026.1766687

**Published:** 2026-02-18

**Authors:** Ludwing Jose Gallo-Motta, Angela Maria Lince-Gonzalez, Diana Lucia Bravo-Guerra, Carlos Alberto Pardo Gonzalez, Rodrigo Perez-Morales, Juan D. Roa G

**Affiliations:** 1 Pediatric Intensivist, Fundación HOMI, Bogotá, Colombia; 2 Pediatric Hematologist-Oncologist, Fundación HOMI, Bogotá, Colombia; 3 Pediatric Neurologist, Pediatric Intensivist, Fundación HOMI, Bogotá, Colombia

**Keywords:** acute lymphoblastic leukemia, pediatric intensive care, PELOD-2 score, phoenix criteria, sepsis

## Abstract

**Introduction:**

Sepsis in children with acute lymphoblastic leukemia (ALL) is associated with high mortality and a frequent need for advanced organ support; therefore, reliable scoring systems are required to stratify risk in the pediatric intensive care unit (PICU).

**Objective:**

To compare the performance of the Phoenix-8 and PELOD-2 scales, measured at 24 and 72 h after admission, for predicting 28-day mortality in children with ALL and sepsis or septic shock.

**Methods:**

A retrospective cohort study including 61 patients aged 1 month to 18 years with ALL admitted to the PICU of a quaternary-level hospital in Bogotá, Colombia, between 2022 and 2024. Phoenix-8 and PELOD-2 scores were calculated at 24 and 72 h after admission. Receiver operating characteristic (ROC) curves, optimal cutoff points, sensitivity, and specificity were analyzed.

**Results:**

Twenty-eight-day mortality was 23.0%; 27.9% of patients required invasive mechanical ventilation and 63.9% required vasoactive support. At 72 h, a PELOD-2 score ≥7 showed an area under the ROC curve (AUC) of 0.945, with a sensitivity of 92.9% and specificity of 91.1%. A Phoenix-8 score ≥8 achieved an AUC of 0.976, with a sensitivity of 92.9% and specificity of 91.3%, and was significantly associated with the use of mechanical ventilation, vasoactive agents, and renal replacement therapy.

**Discussion:**

Phoenix-8 and PELOD-2 demonstrated moderate discriminative ability at admission but excellent performance at 72 h, making them clinically useful and comparable tools for prognostic stratification in children with ALL and sepsis.

## Introduction

Sepsis is defined as life-threatening organ dysfunction caused by a dysregulated host response to infection and remains a major cause of morbidity and mortality in the pediatric population (1). Each year, approximately 7,000 children die from sepsis in the United States, and across different series, mortality from pediatric septic shock ranges between 8.5% and 15.1%, with up to half of deaths occurring within the first 48 h of admission to the pediatric intensive care unit (PICU) (2, 3). A Latin American cohort showed that admission for septic shock is associated with an almost threefold higher risk of death compared with other critical diagnoses (4). In this context, the recent international consensus on pediatric sepsis proposed the Phoenix criteria as a new reference standard for the definition of sepsis and septic shock, integrating organ dysfunction and clinical context based on large databases and expert consensus (1, 5).

In Colombia, childhood cancer represents a priority public health problem. In 2022, 7,748 prevalent cases and 995 incident cases of pediatric cancer were reported, with 403 deaths; acute lymphoblastic leukemia (ALL) accounted for 36.38% of new cases (6). The introduction of more intensive chemotherapy regimens has improved survival; however, this has occurred at the cost of increased rates of profound neutropenia, mucositis, and immunosuppression, which predispose patients to invasive infections, sepsis, and multiple organ dysfunction (7). Approximately 40% of children with cancer require at least one PICU admission during the course of their treatment, with mortality close to 20%, and hematolymphoid neoplasms account for the highest frequency of severe infections, need for intensive support, and risk of death (8–10). In a Colombian series from an exclusively oncologic PICU, sepsis was responsible for nearly 50% of deaths (11). Recent studies have documented that children with cancer and sepsis have a higher risk of death or new morbidity at hospital discharge compared with patients without cancer, even after adjustment for initial severity, underscoring their particular vulnerability (12–14).

Despite the availability of multiple scoring systems to quantify severity and estimate mortality risk in the PICU, their performance in pediatric oncology populations remains suboptimal. Tools designed for the general population, such as PRISM, pSOFA, or the Pediatric Logistic Organ Dysfunction Score (PELOD, updated as PELOD-2), may underestimate or overestimate the true risk in children with hematolymphoid malignancies, due to the interaction between the underlying malignancy, chemotherapy-related toxicity, and the inflammatory response to infection itself (15–17). The Latin American consensus on pediatric sepsis recommends the use of an organ dysfunction score such as PELOD-2 for the initial evaluation and follow-up of patients with sepsis, but acknowledges that evidence specific to pediatric oncology populations is limited (18). Conversely, the Phoenix criteria and their extension, Phoenix-8, which assess eight organ systems, have demonstrated good discriminative ability to define sepsis and predict mortality in general pediatric cohorts; however, evidence in high-risk subgroups such as children with ALL remains scarce and heterogeneous (5, 19). Studies in pediatric patients with cancer have shown that traditional sepsis scores discriminate attributable mortality suboptimally, and although Phoenix and Phoenix-8 appear to improve the prediction of outcomes such as attributable mortality and prolonged PICU stay, their performance is only moderate and with low positive predictive values (12, 20, 21).

Building on this scenario, it is proposed that the particular pathophysiology of ALL—in which disease-related immunodeficiency, chemotherapy-induced myelosuppression, and a high burden of severe infections converge—may modify the behavior of organ dysfunction scoring systems. It is reasonable to hypothesize that a score incorporating immunologic, renal, and hepatic variables, such as Phoenix-8, may more accurately reflect the clinical complexity of these patients compared with scores that assess fewer physiological systems, such as PELOD-2 (7, 13, 14, 16). Our central hypothesis is that, in children with ALL admitted to the PICU with a diagnosis of sepsis or septic shock, the Phoenix-8 and PELOD-2 scales demonstrate different performance in predicting short-term mortality, and that at least one of them will offer a better combination of discrimination and calibration for clinically relevant outcomes.

Accordingly, the primary objective of this study is to compare the ability of the Phoenix-8 and PELOD-2 scales, measured at 24 and 72 h after PICU admission, to predict 28-day mortality in pediatric patients with ALL treated at a quaternary-level pediatric hospital in Bogotá, Colombia. Secondarily, we aim to determine the sensitivity and specificity of each scale, identify clinical and intensive care support factors associated with worse prognosis (such as PICU length of stay, days of mechanical ventilation, use of inotropes and vasopressors, requirement for renal replacement therapy, and other organ dysfunction–related outcomes), and explore the relationship between sepsis severity, as measured by these scores, and in-hospital clinical course (8, 11, 12, 17, 21). Ultimately, this study seeks to generate evidence to support the selection of the most useful scale for early risk stratification in this highly vulnerable population.

## Methods

An observational, retrospective, analytical, and longitudinal cohort study was conducted in the Pediatric Intensive Care Units of the Fundación Hospital Pediátrico de la Misericordia (Bogotá, Colombia). The cohort consisted of children aged 1 month to 18 years with a confirmed diagnosis of acute lymphoblastic leukemia (ALL) who were admitted for sepsis or septic shock between June 1, 2022, and June 1, 2024. The temporal anchor point was the time of PICU admission; from this point onward, clinical and laboratory variables required to calculate prognostic scores and to characterize the clinical course were extracted from the medical records.

The study population was identified from the institutional census of PICU admissions, applying filters based on ICD-10 codes related to sepsis, shock, and ALL, which allowed identification of all eligible events during the study period. Patients of either sex aged between 1 month and 18 years with ALL admitted to the PICU for sepsis or septic shock during the defined dates were included. Exclusion criteria were admission for causes other than sepsis or septic shock, patients with ALL who had not received chemotherapy in the previous 6 months or who were declared in remission, patients with a history of bone marrow transplantation and those with other hematologic malignancies.

The primary outcome was 28-day mortality from the time of PICU admission or until discharge if it occurred earlier. Secondary outcomes included PICU length of stay, use and duration of mechanical ventilation, need for and amount of vasoactive support, and other severity-related events such as renal replacement therapy when applicable. The exposures of interest were the Phoenix-8 and PELOD-2 scores measured at 24 and 72 h after admission. Covariates included age, sex, and a prespecified set of physiological and laboratory measurements that are components of the scoring systems (e.g., lactate, PaFiO₂/SaFiO₂, PaCO₂, creatinine, bilirubin, hematologic counts, Glasgow Coma Scale, mean arterial pressure), as well as the Vasoactive–Inotropic Score (VIS) to quantify the intensity of vasoactive support. All definitions and levels of measurement were previously documented in a data dictionary. The Phoenix-8 version was specifically chosen over the four-organ core because it accounts for renal, hepatic, and immunologic dysfunctions, which are highly prevalent and carry significant prognostic weight in pediatric patients with ALL undergoing intensive chemotherapy.

Data were obtained exclusively from the institutional electronic medical record and laboratory reports. Sepsis and septic shock were defined according to the 2024 International Consensus Criteria. Cases were initially identified using CIE-10 codes, and medical records were subsequently reviewed in a systematic manner to document the presence of suspected or confirmed infection and a Phoenix Sepsis Score of at least 2 points within the first 24 h of PICU admission. This process allowed for a homogeneous characterization of the sepsis cohort included in the study. Phoenix-8 and PELOD-2 scores were calculated using standardized spreadsheets at 24 and 72 h; when available, PaFiO₂ was used, and when unavailable, SaFiO₂ was used according to Phoenix-8 specifications. PELOD-2 and Phoenix-8 scores were calculated for all patients at 24 h after pediatric intensive care unit admission, as complete clinical and laboratory data were available for all 61 included patients at that time point. For the 72-hour assessment, scores were calculated only in patients who remained hospitalized up to that time. Patients who died before 72 h (*n* = 1) were not assigned scores at this second time point. No zero imputation was required for the Phoenix-8 score, as all necessary variables were available. PELOD-2 was calculated only when the minimum required data were present, accounting for the differences in sample size across some 72-hour analyses (Table 6). No patients were excluded due to missing data required for Phoenix-8 calculation. All preprocessing was performed in Excel prior to export to the statistical software.

To minimize bias, homogeneous inclusion and exclusion criteria were defined *a priori*, and time zero was anchored at PICU admission to avoid immortal time bias. Extreme values and missing data were verified against the original source in the medical record; when, despite verification, indispensable information to calculate a score was missing, the record was excluded according to eligibility criteria. To prevent duplication, each patient was assigned a numeric identifier independent of the national identification document. Score calculation was standardized using identical templates and calculation rules for all subjects. In the analysis, confounding was controlled using multivariable models, and collinearity among clinical predictors was explored.

During the study period (June 2022 to June 2024), a total of 539 patients with a diagnosis of acute lymphoblastic leukemia were treated at the institution. Through systematic medical record review, 61 consecutive pediatric intensive care unit admissions for sepsis or septic shock were identified; these cases represent the entire cohort of eligible patients included in the analysis. Given the retrospective design and inclusion of all eligible cases, no formal sample size calculation was performed.

Statistical analysis was performed in two phases. First, variables were described according to their nature and distribution; categorical variables were presented as frequencies and proportions, and continuous variables were presented as mean and standard deviation or median and interquartile range (IQR), selecting the most appropriate measure based on the clinical nature and distribution of each variable. Categorical variables were expressed as absolute frequencies and percentages. Second, bivariate analyses were conducted (e.g., *χ*^2^ test for categorical variables), and for the primary outcome of 28-day mortality, a multivariable logistic regression model with forward selection was fitted, incorporating candidate variables with significant bivariate associations and those deemed clinically relevant. Model performance was assessed using Cox–Snell and Nagelkerke R^2^. Discrimination of the Phoenix-8 and PELOD-2 scores was compared using receiver operating characteristic (ROC) curves, and the standardized mortality ratio (observed/expected) was calculated to contrast observed mortality with that estimated by each score. All analyses were performed using SPSS Statistics version 19, provided by Universidad El Bosque.

## Results

A total of 61 pediatric patients with acute lymphoblastic leukemia admitted to the PICU for sepsis or septic shock were included. Mean age was 9.4 ± 4.2 years, and 57.4% were male. Median PICU length of stay was 5.0 days (IQR 4.0–8.0). Thirteen point one percent of patients received noninvasive mechanical ventilation, 27.9% invasive mechanical ventilation, 63.9% required vasoactive support, and 6.6% required renal replacement therapy. Twenty-eight-day mortality was 23.0%. Regarding severity scores, the median PELOD-2 score was 4.0 (IQR 4.0–6.0) at 24 h and 4.0 (IQR 4.0–7.0) at 72 h, whereas Phoenix-8 showed a mean of 5.2 ± 3.2 points at 24 h and a median of 4.0 (IQR 3.0–8.2) points at 72 h. The maximum VIS score had a mean of 44.5 ± 50.5 (Table 1).

**Table 1:** Characteristics of pediatric patients with acute lymphoblastic leukemia admitted to the PICU for sepsis (*n* = 61).

Variable	Value
Number of patients	61
Demographic characteristics	
Age, years; mean ± SD	9.4 ± 4.2
Sex, *n* (%)	
Male	35 (57.4)
Female	26 (42.6)
PICU support and outcomes	
PICU length of stay, days; median (IQR)	5.0 (4.0–8.0)
Noninvasive mechanical ventilation, *n* (%)	
Yes	8 (13.1)
No	53 (86.9)
Invasive mechanical ventilation, *n* (%)	
Yes	17 (27.9)
No	44 (72.1)
Vasoactive support, *n* (%)	
Yes	39 (63.9)
No	22 (36.1)
Renal replacement therapy, *n* (%)	
Yes	4 (6.6)
No	57 (93.4)
28-day mortality, *n* (%)	
Yes	14 (23.0)
No	47 (77.0)
Days of noninvasive mechanical ventilation^a^; mean ± SD	4.3 ± 4.0
Days of invasive mechanical ventilation^a^; mean ± SD	7.1 ± 5.6
Maximum VIS score^b^; mean ± SD	44.5 ± 50.5
Severity scores	
PELOD-2 at 24 h; median (IQR)	4.0 (4.0–6.0)
PELOD-2 at 72 h; median (IQR)	4.0 (4.0–7.0)
Phoenix-8 at 24 h; mean ± SD	5.2 ± 3.2
Phoenix-8 at 72 h; median (IQR)	4.0 (3.0–8.2)

^a^ Calculated only in patients who received that type of ventilation (NIMV: *n* = 10; IMV: *n* = 16).

^b^ Calculated only in patients who received vasoactive support (*n* = 39).

When analyzing the relationship between severity scores and the use of advanced support, patients who required invasive mechanical ventilation had higher PELOD-2 values at both 24 h (6.0 [IQR 6.0–13.0] vs. 4.0 [IQR 4.0–6.0]) and 72 h (11.0 [IQR 8.0–17.0] vs. 4.0 [IQR 4.0–4.0]), as well as higher Phoenix-8 values at 24 h (7.9 ± 3.7 vs. 4.2 ± 2.3) and at 72 h (11.0 [IQR 9.0–13.0] vs. 3.0 [IQR 3.0–4.0]), with statistically significant differences in all comparisons (*p* < 0.001). Similarly, patients who received vasoactive support showed higher PELOD-2 values at 24 h (6.0 [IQR 4.0–7.0] vs. 4.0 [IQR 2.5–4.0]) and higher Phoenix-8 values at both 24 and 72 h (6.5 ± 3.1 vs. 2.9 ± 1.8 and 5.0 [IQR 3.5–10.0] vs. 3.0 [IQR 2.0–3.0], respectively), with *p* < 0.001 in all comparisons except for PELOD-2 at 72 h, where *p* = 0.005. Among patients who required renal replacement therapy, PELOD-2 and Phoenix-8 values at 72 h were higher than in those who did not require it (PELOD-2 14.0 [IQR 9.2–18.5] vs. 4.0 [IQR 4.0–5.0]; Phoenix-8 11.5 [IQR 10.5–12.5] vs. 4.0 [IQR 3.0–5.2]), with *p* = 0.004 and *p* = 0.006, respectively (Table 2).

**Table 2:** Relationship of PELOD-2 and Phoenix-8 scores with use of advanced support and multiple organ dysfunction (*n* = 61).

Outcome/Group	*n*	PELOD-2 24 h	PELOD-2 72 h	Phoenix-8 24 h	Phoenix-8 72 h
Invasive mechanical ventilation: Yes	17	6.0 (6.0–13.0)	11.0 (8.0–17.0)	7.9 ± 3.7	11.0 (9.0–13.0)
Invasive mechanical ventilation: NO	44	4.0 (4.0–6.0)	4.0 (4.0–4.0)	4.2 ± 2.3	3.0 (3.0–4.0)
Invasive mechanical ventilation: *p* value		<0.001	<0.001	<0.001	<0.001
Vasoactive support: Yes	39	6.0 (4.0–7.0)	4.0 (4.0–10.0)	6.5 ± 3.1	5.0 (3.5–10.0)
Vasoactive support: NO	22	4.0 (2.5–4.0)	4.0 (4.0–4.0)	2.9 ± 1.8	3.0 (2.0–3.0)
Vasoactive support: *p* value		<0.001	0.005	<0.001	<0.001
Renal replacement therapy: Yes	4	7.5 (6.0–10.0)	14.0 (9.2–18.5)	6.8 ± 3.6	11.5 (10.5–12.5)
Renal replacement therapy: NO	57	4.0 (4.0–6.0)	4.0 (4.0–5.0)	5.1 ± 3.2	4.0 (3.0–5.2)
Renal replacement therapy: *p* value		0.042	0.004	0.428	0.006

Regarding the association between severity scores and duration of ventilation, in patients who received noninvasive mechanical ventilation (*n* = 9), Spearman correlation coefficients between days of ventilation and PELOD-2 at 24 and 72 h, as well as Phoenix-8 at 24 and 72 h, ranged from −0.59 to 0.37, without reaching statistical significance (*p* ≥ 0.096). In patients receiving invasive mechanical ventilation (*n* = 16), correlation coefficients ranged from −0.49 to −0.14 across scores and time points, with *p* values between 0.053 and 0.612 (Table 3).

**Table 3:** Correlation between severity scores and days of ventilation.

Outcome (days of ventilation)	*n*	PELOD-2 24 h	PELOD-2 72 h	Phoenix-8 24 h	Phoenix-8 72 h
Total days of NIMV (only patients with NIMV)	9	*ρ* 0.37 (*p* = 0.323)	*ρ* −0.59 (*p* = 0.096)	*ρ* 0.05 (*p* = 0.894)	*ρ* −0.36 (*p* = 0.337)
Total days of IMV (only patients with IMV)	16	*ρ* −0.37 (*p* = 0.163)	*ρ* −0.49 (*p* = 0.053)	*ρ* −0.14 (*p* = 0.612)	*ρ* −0.30 (*p* = 0.253)

With respect to the relationship between sepsis severity and PICU length of stay, PELOD-2 at 24 and 72 h showed Spearman correlation coefficients of 0.126 (*p* = 0.335) and 0.166 (*p* = 0.210), respectively. For Phoenix-8, correlation coefficients were 0.281 (*p* = 0.028) at 24 h and 0.268 (*p* = 0.038) at 72 h (Table 4).

**Table 4:** Correlation between sepsis severity (PELOD-2 and Phoenix-8 scores) and PICU length of stay.

Severity score	*n* ^a^	Spearman *ρ* with PICU days	*p*
PELOD-2 24 h	61	0.126	0.335
PELOD-2 72 h	59	0.166	0.210
Phoenix-8 24 h	61	0.281	0.028
Phoenix-8 72 h	60	0.268	0.038

^a^ *n*: number of patients with complete information for the score and PICU days.

In the bivariate analysis of factors associated with mortality, no significant differences were observed in age or PICU length of stay between survivors and nonsurvivors. The maximum VIS score was higher among patients who died (62.5 [IQR 46.5–116.2] vs. 20.0 [IQR 10.0–23.0]; *p* < 0.001). PELOD-2 values were higher in nonsurvivors at both 24 h (6.0 [IQR 4.0–12.2] vs. 4.0 [IQR 4.0–6.0]; *p* = 0.017) and 72 h (11.5 [IQR 8.5–17.8] vs. 4.0 [IQR 4.0–4.0]; *p* < 0.001). Similarly, Phoenix-8 scores were higher among nonsurvivors at 24 h (5.5 [IQR 4.0–11.8] vs. 4.0 [IQR 3.0–6.0]; *p* = 0.031) and at 72 h (11.0 [IQR 10.0–13.0] vs. 3.0 [IQR 3.0–5.0]; *p* < 0.001). No differences were found in sex distribution or use of noninvasive mechanical ventilation. The proportion of patients receiving invasive mechanical ventilation, vasoactive support, and renal replacement therapy was higher among nonsurvivors, with *p* < 0.001 for invasive mechanical ventilation, *p* = 0.004 for vasoactive support, and *p* = 0.002 for renal replacement therapy (Table 5).

**Table 5:** Factors associated with worse prognosis (mortality) in children with ALL and sepsis (*n* = 61).

Variable	Survivors (*n* = 47)	Nonsurvivors (*n* = 14)	*p*
Age, years	8.0 (6.0–12.0)	10.5 (8.2–13.5)	0.331
PICU length of stay, days	5.0 (4.0–7.5)	4.5 (4.0–10.2)	0.69
Total days of noninvasive MV	3.0 (2.5–10.0)	1.5 (1.2–1.8)	0.094
Total days of invasive MV	5.0 (4.0–7.0)	5.0 (4.0–8.0)	1
Maximum VIS score	20.0 (10.0–23.0)	62.5 (46.5–116.2)	<0.001
PELOD-2 24 h	4.0 (4.0–6.0)	6.0 (4.0–12.2)	0.017
PELOD-2 72 h	4.0 (4.0–4.0)	11.5 (8.5–17.8)	<0.001
Phoenix-8 24 h	4.0 (3.0–6.0)	5.5 (4.0–11.8)	0.031
Phoenix-8 72 h	3.0 (3.0–5.0)	11.0 (10.0–13.0)	<0.001
Género: Female	20 (42.6%)	6 (42.9%)	1
Male	27 (57.4%)	8 (57.1%)	
Noninvasive MV: No	41 (87.2%)	12 (85.7%)	1
Noninvasive MV: Yes	6 (12.8%)	2 (14.3%)	
Invasive MV: No	43 (91.5%)	1 (7.1%)	<0.001
Invasive MV: Yes	4 (8.5%)	13 (92.9%)	
Vasoactive/inotropic support: No	22 (46.8%)	0 (0.0%)	0.004
Vasoactive/inotropic support: Yes	25 (53.2%)	14 (100.0%)	
Renal replacement therapy: No	47 (100.0%)	10 (71.4%)	0.002
Renal replacement therapy: Yes	0 (0.0%)	4 (28.6%)	

When evaluating the performance of the scores for mortality prediction, the optimal cutoff point for PELOD-2 at 24 h was ≥9 points, with an area under the ROC curve (AUC) of 0.706, sensitivity of 42.9%, and specificity of 93.6%. At 72 h, a PELOD-2 cutoff ≥7 points was associated with an AUC of 0.945, sensitivity of 92.9%, and specificity of 91.1%. For Phoenix-8, the optimal cutoff at 24 h was ≥11 points (AUC 0.691, sensitivity 35.7%, specificity 97.9%), and at 72 h ≥8 points (AUC 0.976, sensitivity 92.9%, specificity 91.3%) (Table 6).

**Figure 1:**
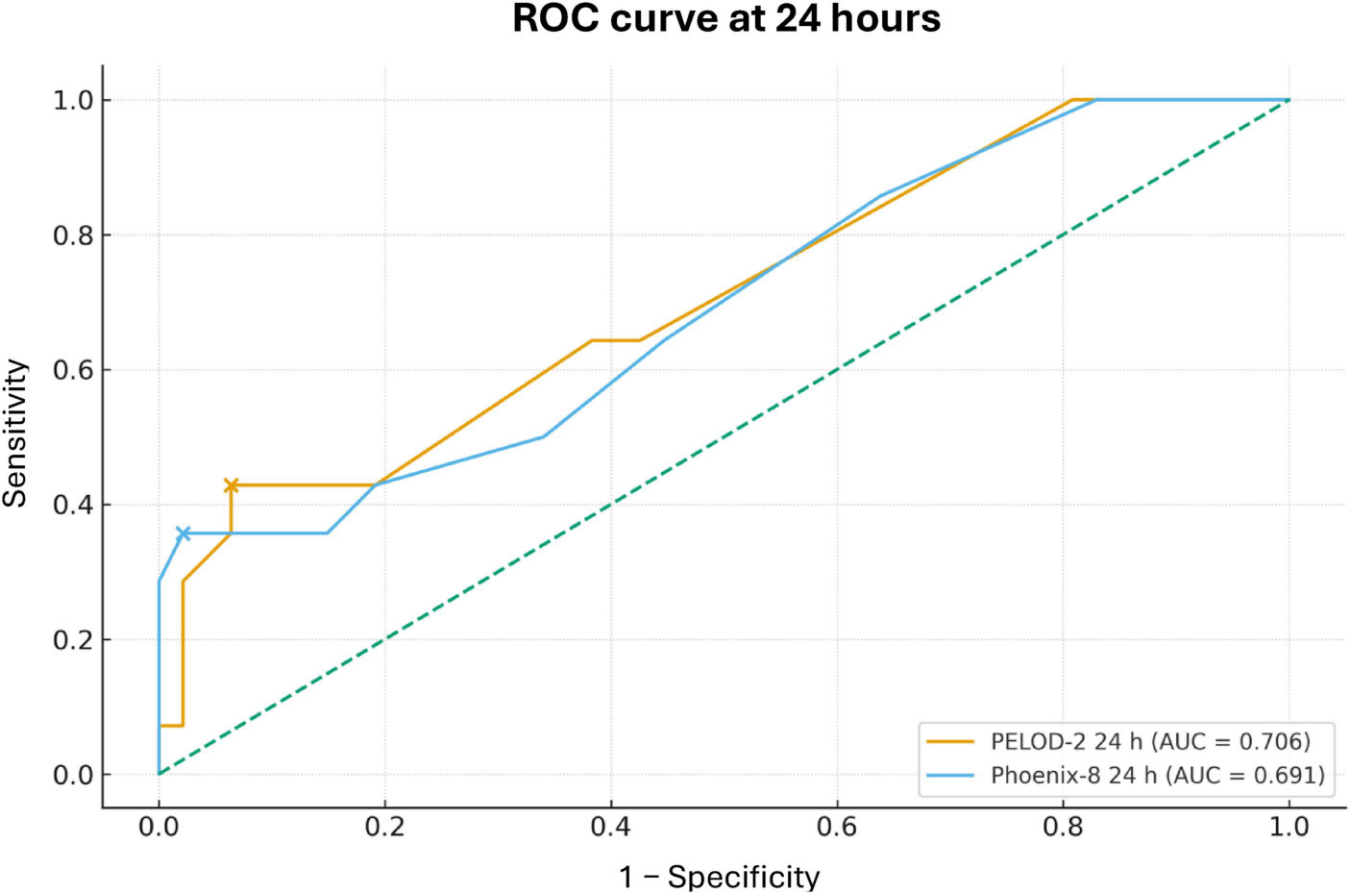
ROC curves of PELOD-2 and Phoenix-8 at 24 h for prediction of 28-day mortality.

**Figure 2:**
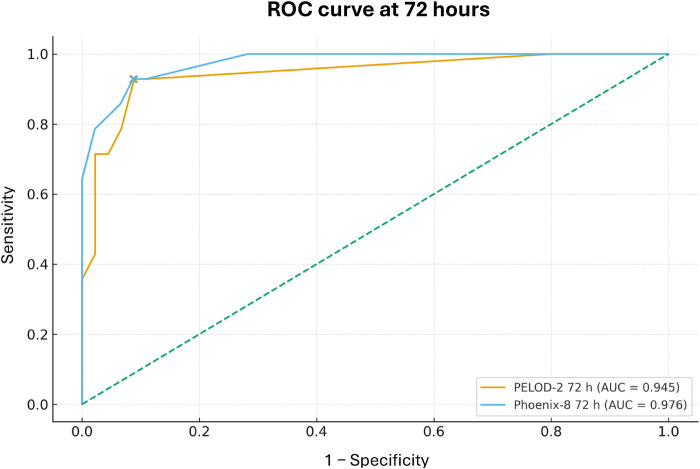
ROC curves of PELOD-2 and Phoenix-8 at 72 h for prediction of 28-day mortality.

Finally, when comparing the overall discriminative ability of the scores, PELOD-2 at 24 h achieved an AUC of 0.652 (95% CI 0.317–0.966) and Phoenix-8 at 24 h an AUC of 0.724 (95% CI 0.383–0.975). At 72 h, the AUC for PELOD-2 was 0.902 (95% CI 0.810–0.980) and for Phoenix-8 0.906 (95% CI 0.814–0.983) (Table 7; Figures 1, 2).

**Table 6:** Sensitivity and specificity of PELOD-2 and Phoenix-8 scores for predicting mortality.

Score	*n* ^a^	Cutoff (≥)	AUC	Sensitivity (%)	Specificity (%)
PELOD-2 24 h	61	≥ 9	0.706	42.9	93.6
PELOD-2 72 h	59	≥ 7	0.945	92.9	91.1
Phoenix-8 24 h	61	≥ 11	0.691	35.7	97.9
Phoenix-8 72 h	60	≥ 8	0.976	92.9	91.3

^a^ *n*: number of patients with complete information for the score and the outcome (mortality).

**Table 7:** Comparison of the ability of PELOD-2 and Phoenix-8 at 24 and 72 h to predict 28-day mortality.

Scale	*n* ^a^	AUC	IC 95% AUC
PELOD-2 24 h	61	0.652	0.317–0.966
PELOD-2 72 h	59	0.902	0.810–0.980
Phoenix-8 24 h	61	0.724	0.383–0.975
Phoenix-8 72 h	60	0.906	0.814–0.983

^a^ *n*: number of patients with complete information for the scale at that time point and 28-day mortality.

AUC, area under the ROC curve. 95% CI obtained via bootstrap resampling.

## Discussion

In this cohort of 61 children with acute lymphoblastic leukemia admitted to the PICU of a quaternary-level hospital for sepsis or septic shock, 28-day mortality was 23%, with a high use of advanced organ support: 27.9% required invasive mechanical ventilation, 63.9% vasoactive support, and 6.6% renal replacement therapy. Nonsurvivors exhibited a greater burden of organ dysfunction, reflected by significantly higher PELOD-2 and Phoenix-8 scores, as well as higher VIS values. The discriminative ability of both scores to predict mortality was limited at 24 h (AUC 0.652 for PELOD-2 and 0.724 for Phoenix-8), but clearly very good at 72 h (AUC 0.902 for PELOD-2 and 0.906 for Phoenix-8), with optimal cutoff points of ≥7 and ≥8, respectively, yielding sensitivities of 92.9% and specificities of approximately 91%. These findings suggest that dynamic reassessment of organ dysfunction during the first 72 h of PICU admission allows better risk stratification for mortality in children with ALL and sepsis, and that both PELOD-2 and Phoenix-8 are useful and comparable tools in this onco-critical care context. It is important to note that the Phoenix criteria were developed using elements from established organ dysfunction scores, including PELOD-2. Specifically, there is an inherent overlap in the cardiovascular and neurologic domains, as both scales utilize similar physiological thresholds (e.g., mean arterial pressure and Glasgow Coma Scale) to define dysfunction. Despite this partial overlap, this study compares their performance to determine if the additional variables and scoring structure of Phoenix-8 offer superior prognostic value in the oncology population.

The mortality observed in our series (23%) falls within the range reported for pediatric sepsis in the PICU and for high-risk oncology populations. In a cohort study from a low-resource hospital, mortality among children with sepsis in the PICU reached 57.9%, reflecting the impact of limited resources and high organ dysfunction burden (22). In contrast, series from high-income countries report overall pediatric sepsis mortality rates in the PICU between 5% and 20% (1). In the subgroup of oncology patients, mortality is consistently higher: Saeed et al. described 63 children with oncologic diagnoses and sepsis admitted to the PICU in Pakistan, of whom only 52.4% survived, corresponding to a mortality of nearly 47.6% (23). Bhosale et al, in India, reported an in-hospital mortality of 26.5% among 200 critically ill pediatric oncology patients, with significantly higher rates in those with hematologic malignancies and those requiring mechanical ventilation and vasoactive support (8). Other studies in pediatric patients with cancer and sepsis or septic shock admitted to the PICU have reported mortality rates ranging from 17% to 41%, particularly when neutropenia, multiple organ dysfunction, and the need for ventilatory and vasoactive support coexist (24, 25). In this context, the 23% mortality observed in our homogeneous ALL cohort, despite the high use of invasive ventilation (92.9% of nonsurvivors) and vasoactive agents (100% of nonsurvivors), suggests that the combination of specialized intensive care and earlier identification of organ dysfunction may be mitigating the traditionally ominous prognosis of sepsis in pediatric patients with hematolymphoid malignancies (24, 25).

Our findings support the role of advanced support intensity as a relevant prognostic marker, in agreement with the literature. In the present study, invasive mechanical ventilation, vasoactive support, and renal replacement therapy were strongly associated with mortality, and nonsurvivors exhibited markedly higher maximum VIS scores (median 62.5 vs. 20 in survivors). This is consistent with the findings of Saeed et al, who reported that multiple organ dysfunction and mechanical ventilation were independently associated with death in pediatric oncology patients with sepsis (24), as well as with more recent series in pediatric critical oncology in which the need for ventilation, inotropes, and a greater number of failing organs remain key determinants of mortality (8, 24). Similarly, in the cohort reported by Rusmawatiningtyas et al. in 2021, conducted in a low-resource setting, mechanical ventilation, vasoactive drug use, and fluid overload greater than 10% were associated with mortality in pediatric sepsis (22), which aligns with our observation that children with ALL who die are those who concentrate the greatest intensity of critical support. These parallels reinforce the external validity of our findings, although they are centered on a single neoplastic entity.

Regarding the performance of organ dysfunction scores, our results are concordant with, and in some aspects superior to, those previously described for PELOD-2 in general PICU populations. Leteurtre et al, in the update of PELOD-2, demonstrated excellent discrimination for mortality in the PICU, with AUC values around 0.93 in general pediatric critical care cohorts (16). A subsequent meta-analysis including 29 studies reported an overall sensitivity of 0.78 (95% CI 0.71–0.83) and specificity of 0.75 (95% CI 0.68–0.81) for PELOD-2 in predicting mortality, with a pooled AUC of 0.83 (16). In our cohort of children with ALL and sepsis, PELOD-2 at 72 h achieved an AUC of 0.902, with sensitivity of 92.9% and specificity of 91.1% for a cutoff ≥7, values clearly higher than pooled estimates from the general population and reflective of the high risk concentration in this oncologic subgroup. However, at 24 h the AUC was only 0.652, with sensitivity of 42.9% for a cutoff of 9, suggesting that in immunocompromised patients, organ dysfunction assessed by PELOD-2 acquires true discriminative power when its evolution over the first days of illness is considered, rather than relying on a single measurement at admission. This dynamic behavior is consistent with studies showing that serial PELOD/PELOD-2 measurements improve prognostic performance compared with a single determination (16).

With respect to the Phoenix criteria, our findings align with recent evidence supporting their adoption as a new standard for defining pediatric sepsis and septic shock. The Society of Critical Care Medicine Task Force demonstrated that a Phoenix Sepsis Score ≥2 in children with suspected infection identifies a group with significantly higher hospital mortality (7.1% in high-resource settings and 28.5% in low-resource settings), increasing risk by more than eightfold compared with infected children not meeting these criteria (1). In the multicenter validation of the Phoenix criteria, Sánchez-Pinto et al. showed that in more than 6,000 children, the Phoenix Sepsis Score calculated within the first 24 h was robustly associated with in-hospital mortality and with the composite outcome of death or ECMO requirement within 72 h, with sensitivities ranging from 55% to 78% depending on the outcome, albeit with modest positive predictive values (5). In our homogeneous ALL cohort, Phoenix-8 at 72 h demonstrated an AUC of 0.906 and, with a cutoff ≥8, a sensitivity of 92.9% and specificity of 91.3%, suggesting that in this high-risk oncologic subgroup, the Phoenix criteria not only retain but enhance their discriminative ability when early organ dysfunction trajectory is incorporated. Conversely, at 24 h the AUC was 0.724 and sensitivity 35.7% for a cutoff of 11, indicating that, similar to PELOD-2, cutoffs derived from general populations may not be directly extrapolatable to immunosuppressed oncology patients when the goal is to maximize sensitivity without excessively sacrificing specificity (16).

Specifically in children with cancer, Wolf et al. reported that the Phoenix Sepsis Score accurately classified the risk of attributable mortality and PICU length of stay in oncology patients with suspected infection, numerically outperforming other scores such as PELOD-2 and pSOFA for attributable mortality, albeit with low specificity at predefined cutoffs (12). Our results extend these observations by demonstrating that in children with ALL and sepsis, Phoenix-8 calculated at 72 h behaves similarly to PELOD-2 in terms of AUC, but with very high sensitivity and specificity for a relatively simple cutoff (≥8), making it an attractive candidate for risk stratification in onco-critical patients. Conversely, Wittmann Dayagi et al. have emphasized that prognostic tools designed for the general pediatric population (PELOD-2, PRISM III) may be insufficient in hemato-oncologic patients, advocating for the development of specific scoring systems for this group (5, 25, 26, 27). In this regard, our cohort suggests that both PELOD-2 and Phoenix-8 retain excellent performance if the timing of measurement (72 h) and cutoffs are recalibrated; however, the very high AUC and sensitivity estimates in a small sample raise concern for overfitting and reinforce the need for external validation.

This study has important limitations that must be considered when interpreting the findings. First, it is a retrospective, single-center cohort with a small sample size (*n* = 61), exclusively including patients with ALL, which limits generalizability to other hematologic malignancies, solid tumors, or hematopoietic stem cell transplant recipients. Estimation of optimal cutoffs using ROC curves in a small sample may overestimate AUC, sensitivity, and specificity, particularly with a low number of events (14 deaths), and thus these thresholds should be considered exploratory rather than definitive. Additionally, detailed and systematic information on key variables such as disease status (new diagnosis vs. relapse), chemotherapy phase, intensity of immunosuppression, presence of prolonged neutropenia, or microbiological characteristics of infections was not available, all of which have been associated with adverse outcomes in pediatric oncologic sepsis (27, 28). Functional outcomes and new morbidity associated with sepsis were also not assessed, despite their increasing recognition as relevant endpoints, especially in children with cancer (27). Finally, although scores were calculated at 24 and 72 h, more complex dynamic models (e.g., serial trajectories of PELOD-2/Phoenix or combinations with biomarkers) were not explored, nor was performance compared with other scores such as PIM-3 or PRISM IV.

An additional limitation is that the Phoenix-8 score was calculated as a cross-sectional measurement at 24 and 72 h, rather than as the maximum value observed within those time windows, as proposed in its original development. Although this approach reflects clinically relevant reassessment time points, it may limit direct comparability with other studies and may have influenced the observed temporal evolution of the scores.

Among the strengths of this study is its focus on a highly homogeneous and very high-risk population—children with ALL and sepsis admitted to the PICU—which reduces clinical variability and allows more precise interpretation of organ dysfunction score performance. The systematic collection of clinical data, organ support variables, and hard outcomes (28-day mortality), along with the head-to-head comparison of PELOD-2 and Phoenix-8 in the same patient cohort, provides original information, particularly in the Latin American context, where studies in critically ill pediatric oncology patients remain scarce (28). Additionally, the use of AUC with confidence intervals obtained via bootstrap resampling, exploration of specific cutoffs, and analysis of the relationship between scores and advanced support use (invasive ventilation, vasoactive agents, renal replacement therapy) strengthen the internal robustness of the findings and their immediate clinical relevance for PICU practice.

From a clinical practice perspective, our results support the routine use of PELOD-2 and Phoenix-8 as complementary risk stratification tools in children with ALL and sepsis, with particular emphasis on reassessment at 72 h. A PELOD-2≥7 or Phoenix-8≥8 at that time point may help identify patients at higher risk of death, justifying intensified monitoring, optimization of hemodynamic and ventilatory support, early consideration of rescue therapies (e.g., ECMO in centers with availability), and timely discussions regarding goals of care when overall prognosis is very poor (5, 16). Nevertheless, these scores should always be integrated with clinical judgment and the trajectory of the underlying oncologic disease, and should not be used in isolation to determine futility or escalation of care. From a research standpoint, our findings underscore the need for multicenter, ideally prospective, studies to validate and recalibrate PELOD-2 and Phoenix cutoffs in diverse hemato-oncologic populations, incorporate functional and quality-of-life outcomes, and explore prognostic models specifically tailored to children with cancer, as proposed by Wittmann Dayagi et al. and other onco-critical care groups (16, 26, 28).

## Conclusion

In this cohort of children with acute lymphoblastic leukemia and sepsis or septic shock, 28-day mortality was high (23%) and concentrated among those with greater need for advanced organ support and higher organ dysfunction scores. PELOD-2 and Phoenix-8 demonstrated only moderate discriminative ability at 24 h, but excellent performance at 72 h, with AUCs close to 0.90 and sensitivities and specificities exceeding 90% for simple cutoff points (≥7 for PELOD-2 and ≥8 for Phoenix-8). These findings suggest that dynamic assessment of organ dysfunction during the first 72 h of PICU admission allows better identification of children with ALL and sepsis at highest risk of death, and support the use of PELOD-2 and Phoenix-8 as useful tools for prognostic stratification in pediatric onco-critical care. However, the single-center design and small sample size warrant interpreting these results as exploratory, highlighting the need for larger multicenter studies to validate these cutoffs and assess their real impact on clinical decision-making and outcomes in children with cancer and sepsis.

## Data Availability

The original contributions presented in the study are included in the article/Supplementary Material, further inquiries can be directed to the corresponding author/s.
